# The Implementation of Neural Networks for Polymer Mold Surface Evaluation

**DOI:** 10.3390/mi15010102

**Published:** 2024-01-05

**Authors:** Hana Vrbová, Milena Kubišová, Dagmar Měřínská, Martin Novák, Vladimir Pata, Jana Knedlová, Michal Sedlačík, Oldřich Šuba

**Affiliations:** 1 Faculty of Technology, Tomas Bata University in Zlin, Vavreckova 5669, 760 01 Zlin, Czech Republic; h_vrbova@utb.cz (H.V.); merinska@utb.cz (D.M.); m8_novak@utb.cz (M.N.); pata@utb.cz (V.P.); knedlova@utb.cz (J.K.); msedlacik@utb.cz (M.S.); suba@utb.cz (O.Š.); 2 Centre of Polymer Systems, University Institute, Tomas Bata University in Zlin, Trida T. Bati 5678, 760 01 Zlin, Czech Republic

**Keywords:** surface quality, roughness parameters, nonlinear regression, perceptron, neural network

## Abstract

This paper presents the measurement and evaluation of the surfaces of molds produced using additive technologies. This is an emerging trend in mold production. The surfaces of such molds must be treated, usually using laser-based alternative machining methods. Regular evaluation is necessary because of the gradually deteriorating quality of the mold surface. However, owing to the difficulty in scanning the original surface of the injection mold, it is necessary to perform surface replication. Therefore, this study aims to describe the production of surface replicas for in-house developed polymer molds together with the determination of suitable descriptive parameters, the method of comparing variances, and the mean values for the surface evaluation. Overall, this study presents a new summary of the evaluation process of replicas of the surfaces of polymer molds. The nonlinear regression methodology provides the corresponding functional dependencies between the relevant parameters. The statistical significance of a neural network with two hidden layers based on the principle of Rosenblatt’s perceptron has been proposed and verified. Additionally, machine learning was utilized to better compare the original surface and its replica.

## 1. Introduction

Injection-molded polymer products mainly need to exhibit excellent mechanical and surface properties, which is crucial for visible surfaces. As such, creating a high-quality surface in an injection-molded polymer mold and its evaluation is essential for its production [1].

Surface properties are, however, often determined already during the injection molding process, and the quality of the mold cavity surface is one of the factors influencing the product quality [2]. As such, the surface quality of the final polymer product is predetermined by its mold. Creating a high-quality surface in an injection-molded polymer mold, together with evaluating this surface, is, therefore, fundamental for production [3].

The mold surface also undergoes wear and overall deterioration with continuous use. This mainly depends on the number of injection cycle repetitions and will result in lower-quality surfaces on the polymer products over time [4].

Presently, mold surfaces are often created using laser beam-based methods, which are expensive despite their relatively widespread nature [5]. As such, the current trend is toward the development of polymer molds created through rapid prototyping techniques as a solution. The desired surface of the mold can then be created during the 3D printing process or modified using a laser beam [6].

Because of the complexity and size of such polymer molds, it is difficult or nearly impossible to correctly measure the roughness directly inside the cavity. Therefore, several replicate materials were sold and used. The standard declared error between the replica and the original surface is approximately several micrometers or percents [7]. Verification of this declaration is difficult, as the production of a replica depends not only on the chemical composition and properties of the replication material but also on the replicator’s skill, compliance with replicability conditions, and especially the type of replicated surface [1,8]. The process itself may also have inherent problems. For example, methyl methacrylate casting resin used to be the most commonly used material for surface replication for industrial use in mechanical engineering, electrical engineering, and chemical and metallurgical laboratories. Methyl methacrylate is a known irritant that is harmful to health. Therefore, replication materials based on C-silicones have been used in the production of replica injection molds [9,10].

If replicas are properly made, the characteristics describing their surface properties from a metrological point of view can then be classified as a measurement and evaluation of amplitude, frequency, or hybrid parameters of the so-called surface roughness [9]. However, from a statistical point of view, the parameters characterizing the surface of the original and the replicas are further burdened with different systematic and random errors, which complicates the determination of any parameter. Therefore, it is a standard procedure in scientific practice to use this basic separate approach [1,10]. It is also possible to use a complex solution that based on the measured parameters on the surface replicated “automatically”, finds a suitable spatial model and calculates the parameters of the original surface at a predetermined confidence level [11].

Therefore, for our approach, the most practical solution is to use elements of machine learning, neural networks with hidden layers, utilizing learning techniques based on Rosenblatt perceptrons. Suitable parameters that characterize the original surface after creating the appropriate replica are then determined as a result obtained at the output layer of the neural network [12]. There are two methods by which the accuracy of predictions can be compared. The first is a comparison of the size of the mean error for the data used for learning the neural network and the test data in relation to the number of learning cycles of the neural network. The term data refers to the measured values of the roughness parameters of both types of surfaces. The second way would be to compare the so-called “relative predictability” of the relevant parameters.

In this study, a new innovative method for the evaluation of surfaces and their replicas was developed. After a basic check of the data values of the parameters Ra and Rz, classical statistical tools belonging to the field of “Hypothesis Theory” (Fisher–Snedecor F-test and two-sided *t*-test) were used [13]. The next step is to find the nonlinear regression functions for the parameters Ra and Rz of the original and replicated surfaces. As can be seen in the relevant relations and proofs, these functions are of the same type for both the parameters described and the types of surfaces produced. Only the estimates of the regression coefficients of the respective regression functions differed statistically significantly [14]. This approach is considered by the authors to be crucial because it allows them to assess the quality of the replicated surface relative to the original surface [15]. If the function is of the same type, this is followed by the final construction of the perceptron neural network and the start of its learning process. Thus, only the “one” perceptron neural network is sufficient, which is then “learned” on the data presented by the parameters Ra and Rz, both on the original and replicated surfaces. Thus, there is no need to construct (in the presented case of the four examples) multiple neural networks; only a single network learned on the corresponding parameter and surface type is required.

## 2. Materials and Methods

### 2.1. The Mould

**Figure 1:**
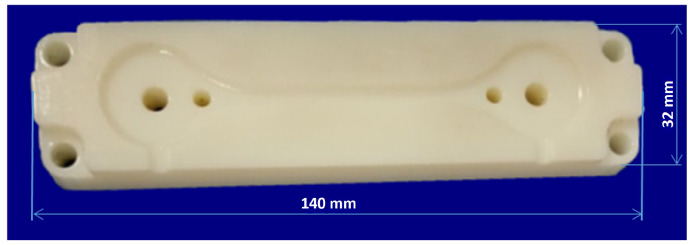
Form printed on a 3D printer.

A 3D printed mold insert with a layer thickness of 25 µm was produced on a Polyjet 3D printer (Rapid Prototyping Objet Eden 250, Treatstock, Newark, DE, USA) and consisted of an acrylic monomer with a photoinitiator (Figure 1). Post-thermal treatment was then provided to increase HDT temperature up to 95 °C with an applied load value of 0.45 MPa. Printing was conducted under the following laboratory conditions: an air temperature of 23 °C, a barometric pressure of 1015 atm, and a humidity of 40%. The mold quality was investigated after 30 molding cycles.

### 2.2. The Material Used for Replication

Two different compositions of impression materials from the Stomaflex^TM^ (Pentron, Jičín, Czech Republic) brand belonging to the C-silicone group were used in the replica production for comparison [9]. This set of materials was selected because of their ability to capture even micrometric surface details. First, a very-high-viscosity-condensing silicone impression material (Plus Putty©) (Pentron, Jičín, Czech Republic) was used. This condensation silicone impression material with a high tolerance to the amount of the catalyst (±40%) allows for excellent application properties, a high value of recovery after deformation, and excellent volume stability. Second, an advanced condensation silicone impression material with a low viscosity and high detail reproduction fidelity, called the Plus Light© (Pentron, Jičín, Czech Republic) type, was utilized.

Finally, a system composed of the classic Plus Putty© and Plus Light© materials with a low viscosity was used. It offers even more detailed reproduction, easy manipulation, and high handling tolerance. A paste catalyst for condensing silicone was used to mix the two materials. Their properties are listed in Table 1 [9].

### 2.3. Production of a Replica of the Original Surface

To ensure replicability, the replication process was designed in accordance with the different handling requirements of the materials used. The replication material used for this experimental set comprised the base material Plus Putty© used for reinforcement, and Stomaflex Plus Light© for the replica itself. This combination has proven to be the most advantageous and has been utilized in previous studies [15]. The replica was manufactured under the following laboratory conditions: an air temperature of 23 °C, a barometric pressure of 1015 hPa, and a humidity of 40%.

**Figure 2:**
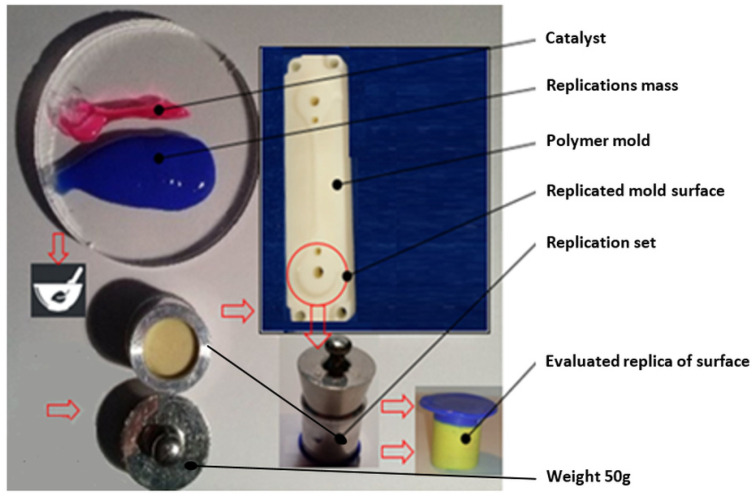
Creating the replica.

The process of manufacturing the replica can be seen in Figure 2.

### 2.4. Optical Microscopy

The examined surfaces of the mold and its replica were evaluated by an optical microscope (Leica DMI 3000 M, Leica Microsystem GmbH, Wetzlar, Germany) at 100× magnification. The 3D images on the studied surfaces were assembled using LAS X 3D visualization software (Leica Microsystems, Germany).

**Figure 3:**
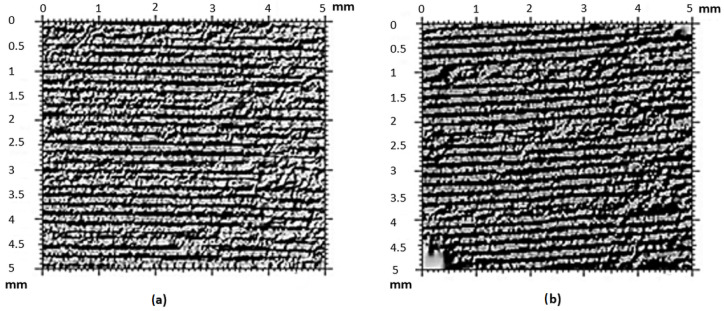
Optical microscope surface control magnified 100×: (**a**) original mold form, (**b**) replica.

This microscope can detect surface defects on a macroscopic scale with fast three-dimensional (3D) imaging. To create 3D images of the surface defects, the samples were scanned in small increments (10 μm). Optical microscopy 2D images of the surface were then recorded after removing a certain thickness (50 μm), as seen in Figure 3.

### 2.5. Optical Surface Scanner

A NewView™ 9000 optical surface scanner (ZYGO™ Middlefield, Middlefield, CT, USA) was used because it provides good versatility in terms of non-contact optical surface profiling. The system allows fast and easy measurement of a wide range of surface types, including smooth, rough, flat, inclined, and staggered surfaces.

The NewView™ 9000 optical surface scanner uses ZYGO’s Mx™ software to provide complete system control and data analysis, including interactive 3D maps, quantitative topographic information, and intuitive measurement navigation. The roughness parameters utilized in this work are the Ra-Arithmetical mean deviation of the assessed profile and the Rz-Maximum height of the assessed profile. These parameters were obtained from both the mold and replica for comparison with the set at a scanning time of 120 s.

## 3. Results

After the creation of replicas, both surfaces had to be checked for surface defects that could have arisen during the replication process or mold printing. For this purpose, optical microscopy was used, specifically a Leica metallographic microscope with 100× optical magnification. Figure 3a shows the original surface of the mold, and Figure 3b presents the replica. A comparison between Figure 3a,b shows that the surface defects are not significant enough to cause deterioration in the replicated surface, rendering it incomparable to the original.

**Figure 4:**
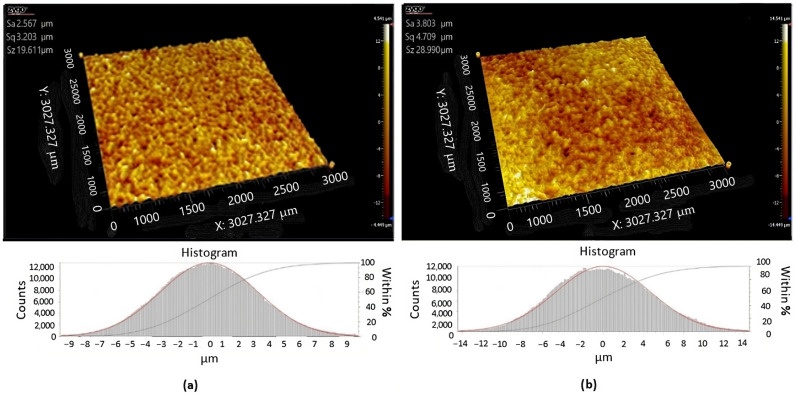
A 3D scan of the (**a**) original mold, (**b**) replica.

Subsequently, both surfaces were scanned using a Zygo New View 9000 profilometer. As shown in Figure 4, the resulting scans were cut using the software (according to ISO 4287 [16]), and the individual sections were then evaluated according to ISO 4288 [17].

An optical filter was used during 3D profilometer scanning to increase the scan accuracy, especially in difficult-to-scan sections of the surfaces of both types. The scan obtained by the 3D profilometer was further processed by the method of least squares at the recommendation of Whitehouse [1] to determine the optimal position of the sections on both surfaces. A 3D spectrum was obtained through the Fast Fourier Transform (FFT) technique [18,19]. The total number of sections processed by this method was n = 100, from which the amplitude parameters of roughness were determined according to the ISO standards mentioned above. A graphical representation of the distribution of the parameters Ra and Rz is shown in Figure 4.

**Figure 5:**
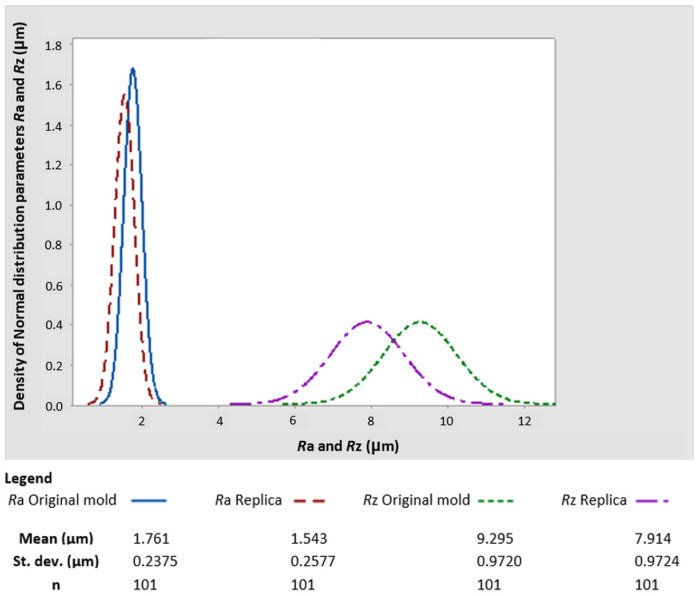
Histogram density of a normal distribution.

Figure 5 also shows that parameters Ra and Rz vary with sufficient statistical significance. In practice, this means that each measured value on the replica and original was burdened with a pair of errors. Specifically, each parameter was burdened with systematic and accidental errors, where the latter type may be eliminated with sufficient measurements.

To evaluate only one of the parameters, elementary statistical tests were conducted using hypothesis testing to compare variances and differences in arithmetic means. This method’s results are presented in Table 2.

As can be seen, each parameter pair had its own original statistical moments and standard deviations. Furthermore, these parameters were functionally linked, and it was impossible to evaluate them separately to maintain objectivity. To prove this assertion, F-test and *t*-test determinations for the Ra and Rz parameters of the original and replicated surfaces were performed.

The results of the variances test are as follows:

*p*-value = 0.416 for the parameter Ra and *p*-value = 0.997 for the parameter Rz. The confidence level of both sets was set to 1 − α = 0.95.

Therefore, it is not possible to reject the agreement of the variances. By contrast, we reject the precise arithmetic average of the test of equality of means. The results are listed in Table 2.

**Figure 6:**
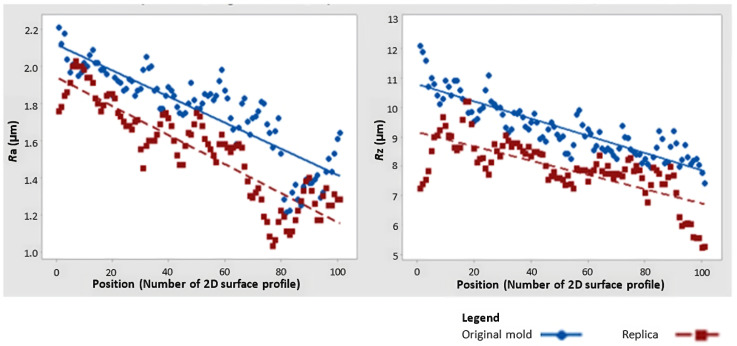
Scatterplot of Ra and Rz: the “Original” mold and its “Replica”.

Following the above procedure, a surface replica was made accordingly. Conversely, the arithmetic means of the amplitude parameters deviate systematically. Therefore, the use of F-tests and *t*-tests is more suitable for the assessment of parameters separately, and not for the global assessment of all surfaces. Therefore, we investigated which parameter to use as a discriminant and utilized the scatterplot shown in Figure 6 for this purpose [20].

**Figure 7:**
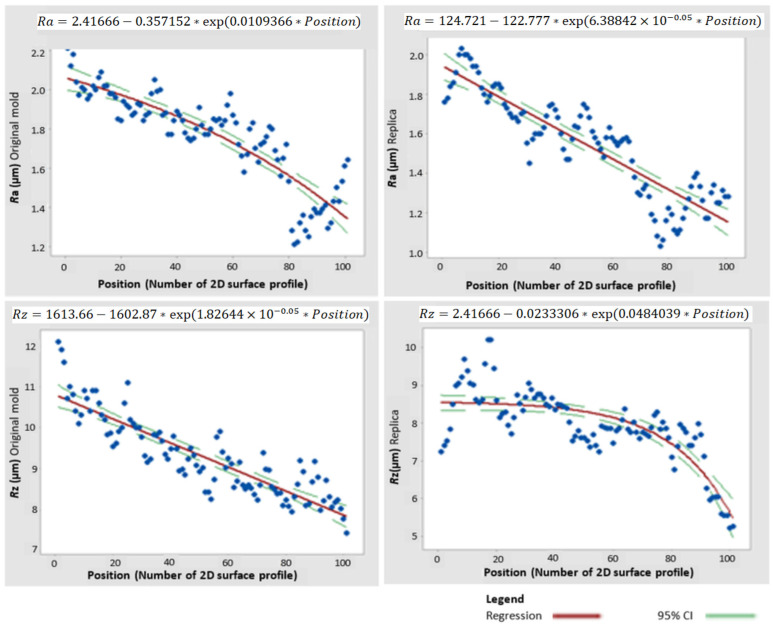
Nonlinear regression functions.

Based on Figure 7, we can assert that the best discriminant parameter will be the parameter Rz as its variance, when comparing the replica with the original, is minor in comparison to the Ra parameter.

Furthermore, it was necessary to identify what the functional dependence between the parameters Ra and Rz in the form of nonlinear regression (Figure 7) [2,21]. Obtained nonlinear regression functions were then investigated using the Levenberg–Marquardt algorithm based on the recommendation of Meloun [2]. Considering a confidence level of 95%, a maximum of 200 iterations and a convergence tolerance of 1–5 were set.

The separately solved parameters, visually represented by the nonlinear regression function, differed only in their regression coefficients. These coefficients must be individually determined for each type of replica and original surface [22].

**Figure 8:**
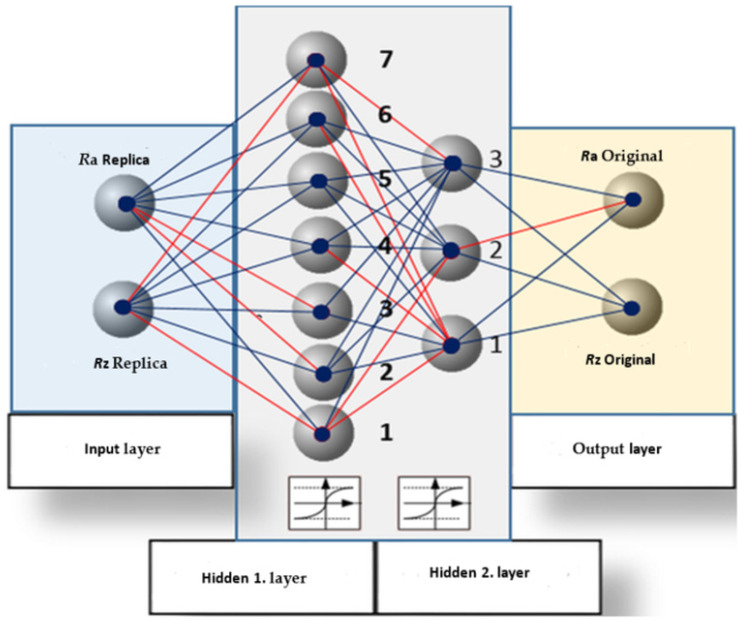
Proposed neural network structure.

To use the described variant of the evaluation process, where the individual amplitude parameters will not be evaluated separately but globally, it is necessary to use a more sophisticated tool: neural networks with hidden layers (Figure 8). This method is based on modeling the relationship between a multidimensional input variable and a multidimensional output variable. These multidimensional variables form the input and output layers of a neural network. The basic philosophy behind this work is that the input amplitude parameters found by the cluster analysis of the replicated surface are introduced into the input layer. Subsequently, the predicted amplitude parameters of the original surface were obtained at the output layer [1,19].

This study aims to determine the number of hidden layers and neurons in these layers. Furthermore, it was necessary to identify the corresponding synapses in the created neural network. To solve this problem, a network using adaptive linear neurons, which can approximate any continuous network, has been successful. Algorithmically, the backpropagation method was used [12,23] to minimize the square of the difference between the actual and expected output. It was also necessary to find an activating differentiable function, which was chosen as a sigmoid function, as per the recommendation [18].

The structure of the neural network is illustrated in Figure 8. Blue indicates positive synapses, and red indicates negative synapses.

The number of iterations was chosen to be 10,000, and the percentage of data for the neural network’s own learning was 70%. As the graph of mean errors between test and learning data shows, it can be stated that the mean error has a gradually decreasing character and takes on the following values.

**Figure 9:**
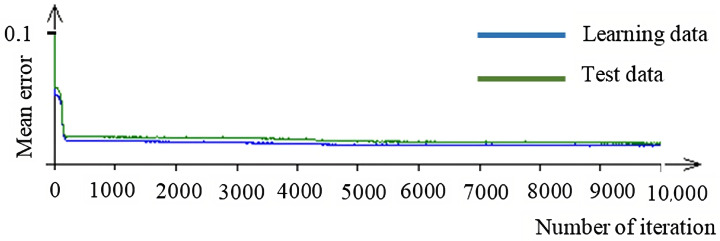
Neural network—medium error.

According to the maximum and mean errors for the learning and testing data presented in Table 3, this neural network can be considered learned. The statistical significance of the found neural network was tested at a confidence level of 0.96 with the result *p* = 3.584443439 × 10^−34^, which confirms the statistical significance of the neural network with a 96% confidence. The mean error for this neural network is shown in Figure 9.

By the “iteration” in this case, we mean a cycle during which the appropriate synapses are created between individual layers of the network occupied by neurons with the help of test and learning data. As can be observed, the network gradually learns, and the mean error difference between the test and learning data gradually decreases. Simultaneously, the mean error curves for individual data types decreased. After 10,000 iterations, the difference was negligible, and the graph was linearized. The neural network can be considered learned and is now ready to solve the above-described problem of the elimination of systematic errors of the surface parameters Ra and Rz.

**Figure 10:**
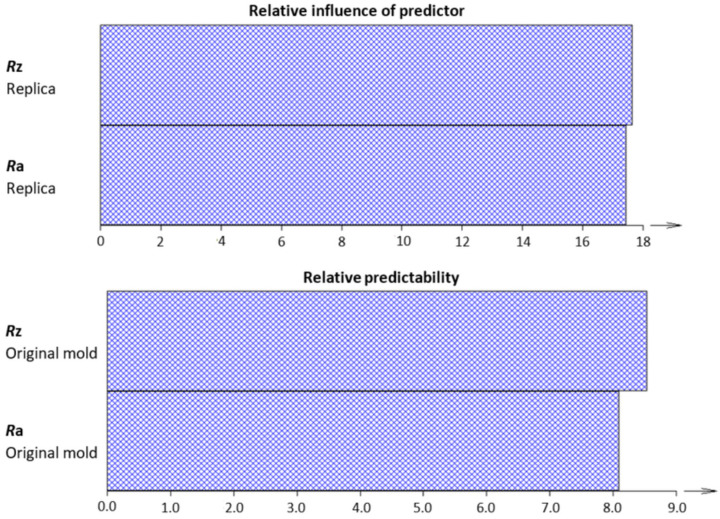
Chart of the relative influence of predictors.

As previously assumed and determined above, the cluster analysis for both the replicated and original surfaces assumes a higher value of the amplitude parameter Ra than Rz. The proof of this is a chart of the relative influence of predictors and relative predictability (Figure 10).

## 4. Discussion

When analyzing the results, it is necessary to note the use of the F-test (more precisely the Fisher–Snedecor F-test) and a two-sided *t*-test to evaluate the Ra and Rz parameters of the replicated and original surfaces [1]. An exploratory analysis, specifically quantile graphs and pie charts, was used for the measured data to confirm that there was no statistically significant asymmetry in the data. Normality tests were then performed at a confidence level of 0.95, according to the Anderson–Darling test and the combined slope and sharpness test [2,24].

Consequently, we cannot reject the hypothesis that the measured data come from a file with a normal distribution, with the possibility of an error of 0.05. To prove that there was no trend or autocorrelation in the data at the same confidence level of 0.95, tests of homogeneity and data independence were necessary.

Because of this necessity, the Fisher–Snedecor F-test followed these preparatory but extremely important tests (see Figure 5). It did not reject the agreement of variances at the confidence level mentioned above. A two-sided *t*-test subsequently confirmed this result. At least in the case of the non-rejection of data normality and non-rejection of equality of data scatter, it measured the amplitude parameters Ra and Rz of the replicated and original surfaces.

This was followed by a search for suitable descriptive amplitude parameters that are often used in scientific studies [1,12]. Time-series plot graphs were first assembled for this purpose. They have shown a specific but undetermined trend in the individual amplitude parameters. This trend was later investigated and quantified using nonlinear regression functions, but only separately for each parameter and surface type.

A multidimensional statistical method, called cluster analysis, was used to search for suitable descriptive parameters. Dendrograms of object similarity were created, and the degree of self-similarity was evaluated according to the so-called cophenetic correlation coefficients. The clustering methodology was tested using single linkage, furthest neighbor, unweighted pair group, weighted pair group, and Ward’s minimum variance method. Ward’s method was then selected as optimal according to the cophenetic correlation coefficient [11,12]. Separate regression functions were also found for each parameter, Ra and Rz, according to the Levenberg–Marquardt method [11].

Classical residues have also been investigated. Specifically, the jackknife residue, Cook distances, and normalized and plausible distances were determined together with function sensitivity maps to determine the quality of the data.

Considering the aforementioned theory, the current trend is not to use separate estimates of individual pairs of amplitude parameters but elements of machine learning, which has already been proven, for example, in an article by Chakrabarti [13]. In that case, the above-designed and learned two-layer neural network based on linear perceptrons was tested for statistical significance at a confidence level of 0.95. Its significance was not rejected, and it had 95% confidence [2].

Graphs of the relative influence of predictors and relative predictability, which, per the dendrogram of cluster analysis, are highlighted as the most important parameter, Rz. Ra also supported the validity of the theory described above [2].

## 5. Conclusions

There is an observable trend in molds produced through rapid prototyping techniques. Their creation and repair often utilize alternative laser beam-based machining methods. Therefore, this work aimed to address the issue of the accuracy of the evaluation of the original surface of the polymer mold created by rapid prototyping technology with the subsequent creation or repair of the pattern.

A comprehensive description of the process of replicating the surface of a polymer mold is provided. A silicone-based Siloflex Plus Putty^®^ replication compound was used; however, our own designed and tested recipe was used to create a mixture specifically for such complicated impressions.

The procedure for finding suitable normative parameters of surface roughness according to ISO 4287 was described and served as a basis for the evaluation of the replicas and the actual form. The procedure to find a suitable method or algorithm that can sufficiently compare the two surfaces in a comprehensive approach instead of separately, as is usual, which was developed by testing hypotheses of variances and means.

Such a procedure is necessary, as it has been demonstrated that the evaluation of scattering and mean values of the original and replicated surface parameters that are often described in the literature can be used only for separate evaluations. This stems from the fact that each parameter is affected by a slew of different random and systematic errors in the creation of the original replica. These problems have been solved by utilizing a designed, learned, and tested neural network based on Rosenblatt’s perceptron. The proposed neural network architecture in this study was tested for amplitude, frequency, and hybrid parameters in both 2D and 3D, according to the relevant ISO standards. The amplitude parameters, Ra and Rz, were based on the most common industrial and scientific requirements, respectively.

Based on our experience, the proposed statistical approach with elements of machine learning can be considered innovative and relatively easy to apply to the technology used in mold production and modification or the repair of their designs.

## Figures and Tables

**Table 1:** Parameters of used replication materials at a temperature of 23 °C.

Tested Property	Stomaflex Plus Putty	Stomaflex Plus Light
Mixing time	Max. 45 s	30 s
Consistency	22.0–26.0 mm	36.0–44.0 mm
Total pot life	Min. 5 s	Min. 100 s
Setting time	2:15–3:00 min	4:00–4:45 min
Recovery after deformation	Min. 97.5%	Min. 98.5%
Linear dimensional change	Better than ǀ0.5ǀ%	Min. ǀ1.3ǀ %

**Table 2:** Test of equality of variances and means.

**Test of Equality of Variances (Fisher–Snedecor F-test)**	
Null hypothesis σ(Ra_Original)/σ(Ra_Replica) = 1 Alternative hypothesis σ(Ra_Original)/σ(Ra_Replica) ≠ 1 Significance level 1 − α = 0.5 *p*-Value = **0.416**	Null hypothesis σ(Rz_Original)/σ(Rz_Replica) = 1 Alternative hypothesis σ(Rz_Original)/σ(Rz_Replica) ≠ 1 Significance level 1 − α = 0.95 *p*-Value = **0.997**
**Test of Equality of Means (Two-Sided *t*-Test)**	
Null hypothesis µ(Ra_Original) = µ(Ra_Replica) = 1 Alternative hypothesis µ(Ra_Original) ≠ µ(Ra_Replica) ≠ 1 Significance level 1 − α = 0.95 *p*-Value = **0.000**	Null hypothesis µ(Rz_Original) = µ(Rz_Replica) = 1 Alternative hypothesis µ(Rz_Original) ≠ µ(Rz_Replica) ≠ 1 Significance level 1 − α = 0.95 *p*-Value = **0.000**

**Table 3:** Table of the maximum and mean errors for learning and testing data.

Maximum and Mean Errors	Confidence Level
Maximum Error for Learning Data	0.0643
Medium error for learning data	0.0097
Maximum error for test data	0.0526
Mean error for test data	0.0152

## Data Availability

Data are contained within the article.
